# Supplementary material for: Chemical Constituents from Albiziae Cortex and Their Ability to Ameliorate Steatosis and Promote Proliferation and Anti-Oxidation In Vitro
Source: Molecules. 2019 Nov 7;24(22):4041. doi: 10.3390/molecules24224041 (PMC6891805; doi:10.3390/molecules24224041)
Supplement: Supplementary file 1 [file molecules-24-04041-s001.pdf]

Supplementary Materials:

Chemical Constituents from *Albiziae Cortex* and Their Ability to Ameliorate Steatosis and Promote Proliferation and Anti-oxidation In Vitro

Xuelin Shi[a,1], Zhongjie Li[a,1], Weiwei Cai[b,1], Yixiao Liu[b], Shuangshuang Li[b], Min Ai[b], Jiangnan Sun[b], Bao Hou[b], Lulu Ni[b,*] and Liying Qiu[b,*]

[a] School of Pharmaceutical Sciences, Jiangnan University, Wuxi 214122 , China.
[b] Department of Basic Medicine, Wuxi School of Medicine, Jiangnan University, Wuxi 214122 , China.
[1] Equal contributors.
* Correspondence: qiulydoc@sina.com (L. Qiu), nllandylau002@163.com (L. Ni).

^{1}HNMR-^{13}CNMR and HR-MS spectra of the compounds **1-5**.

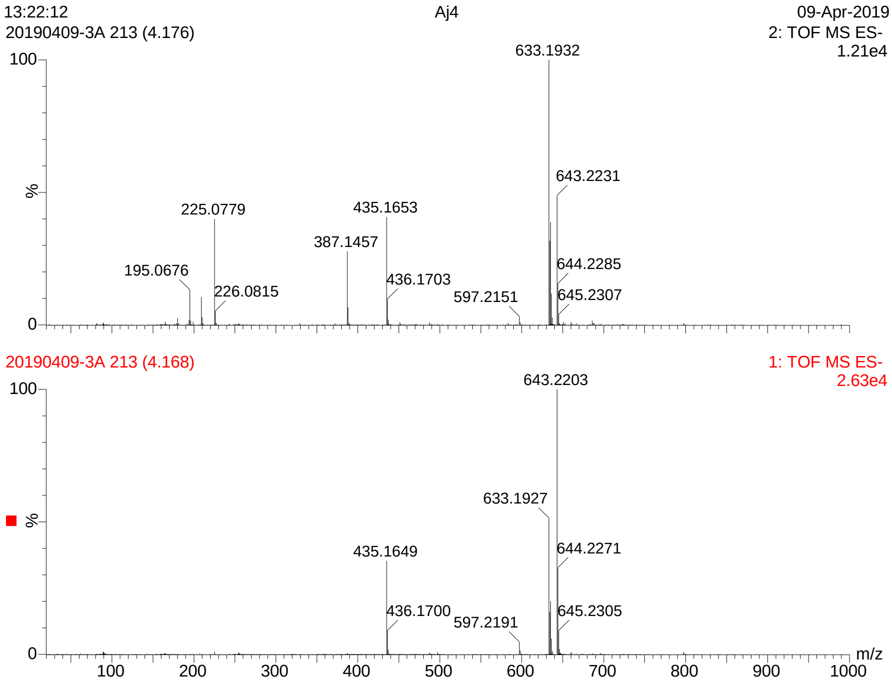

Fig. S1 HR-ESI-MS spectrum of compound **1**.

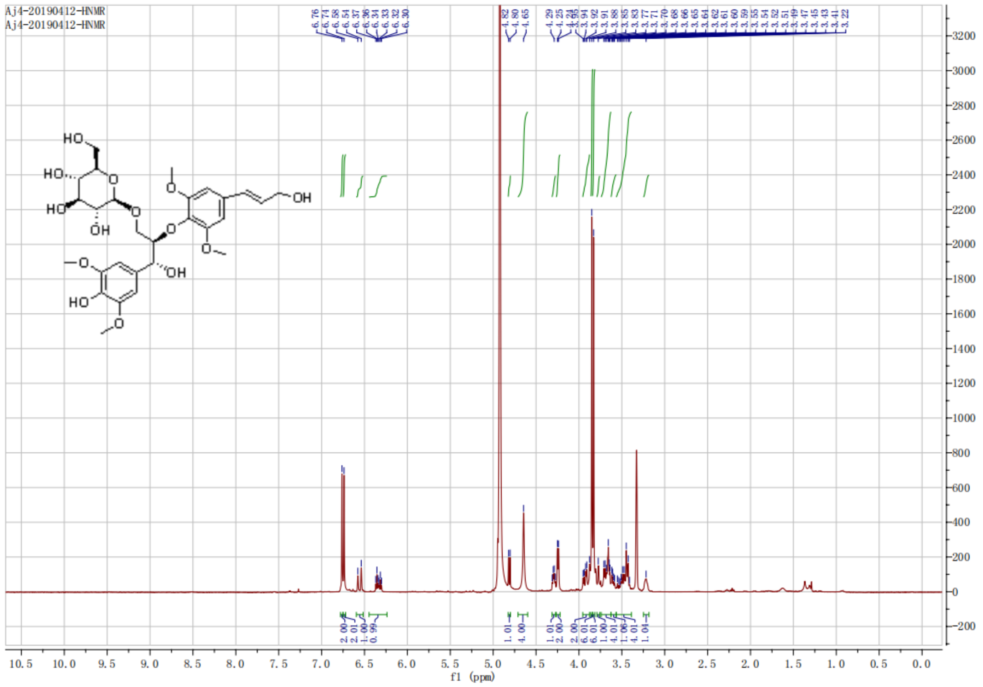

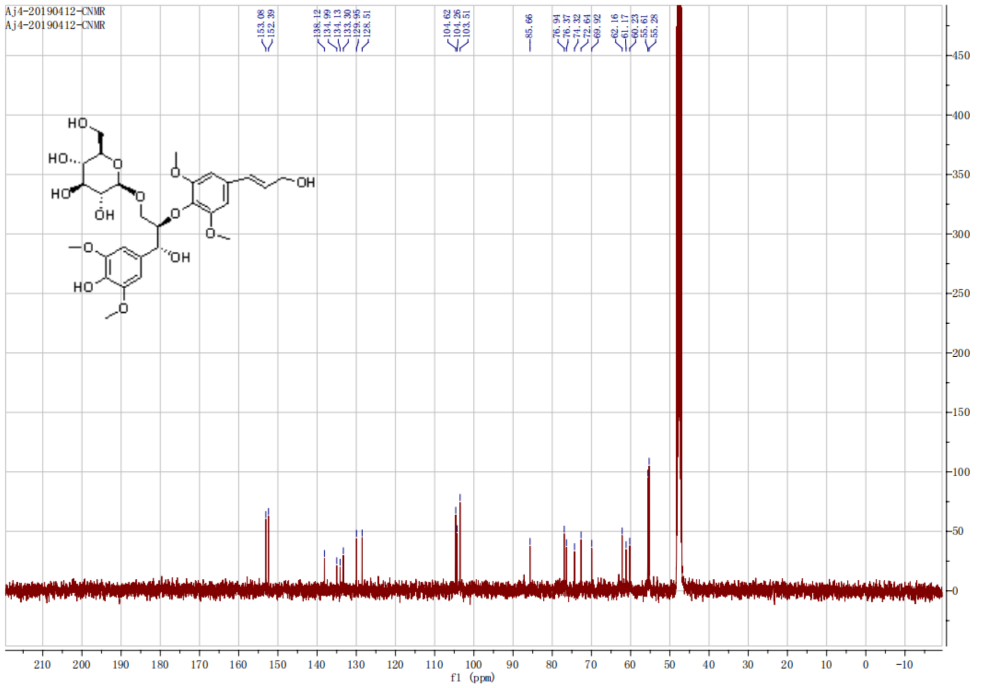

Fig. S2 ^{1}H-^{13}C NMR spectrum of Compound **1**.

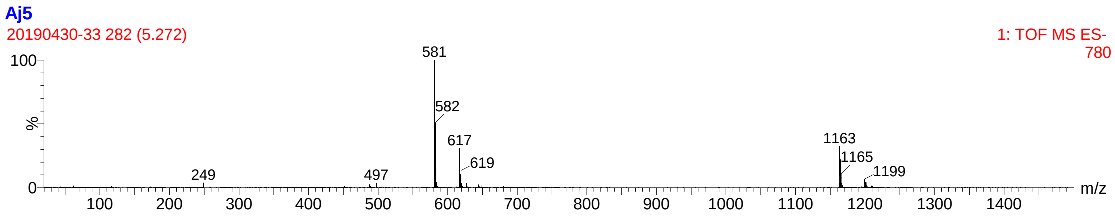

Fig. S3 ESI-MS spectrum of compound **2**.

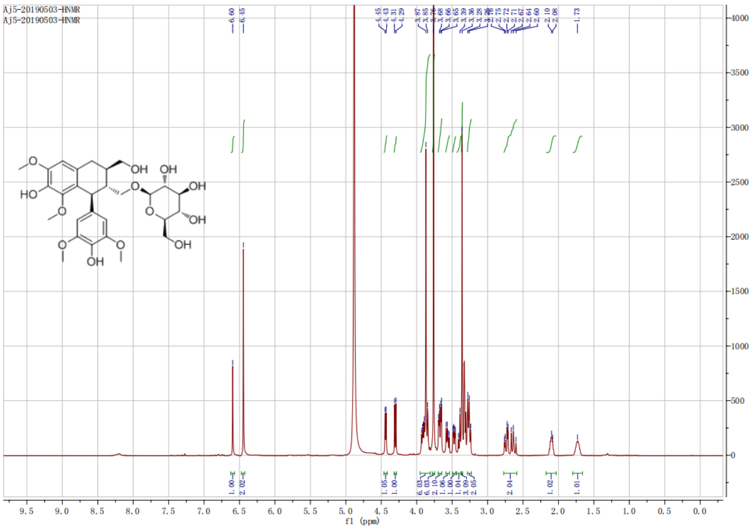

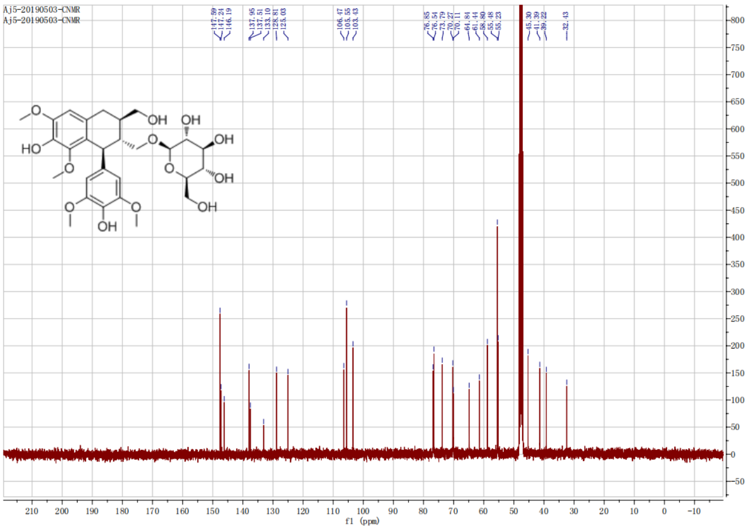

Fig. S4 ^{1}H-^{13}C NMR spectrum of compound **2**.

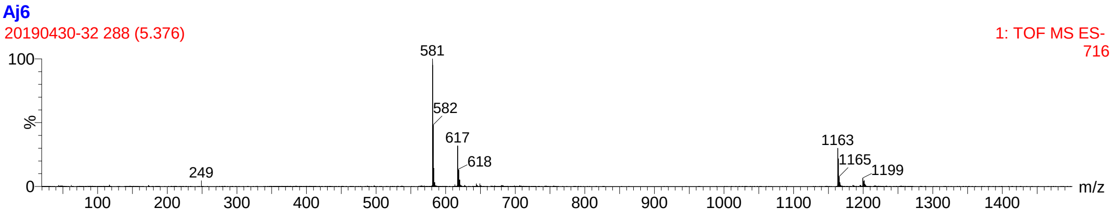

Fig. S5. ESI-MS spectrum of compound **3**.

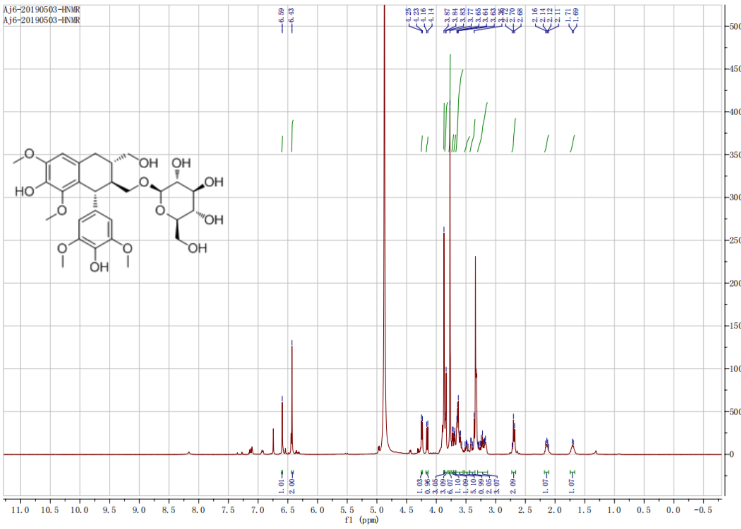

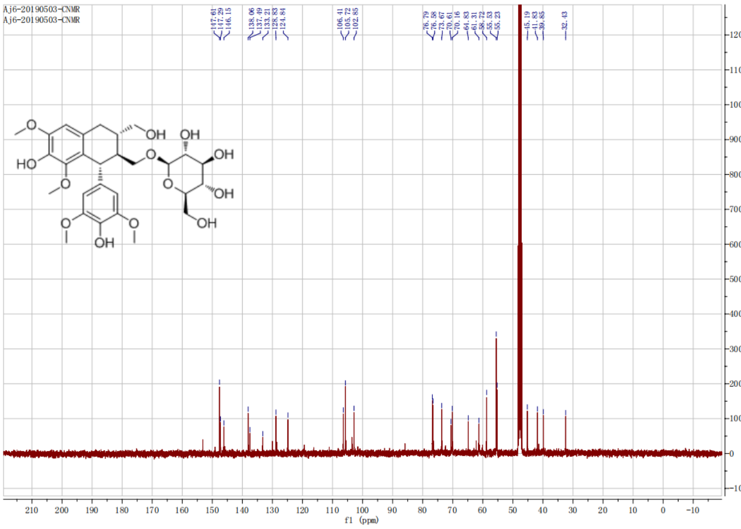

Fig. S6. ^{1}H-^{13}C NMR spectrum of Compound **3**.

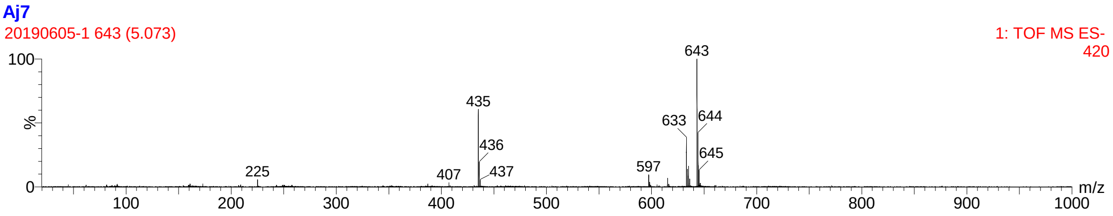

Fig. S7. ESI-MS spectrum of compound **4**.

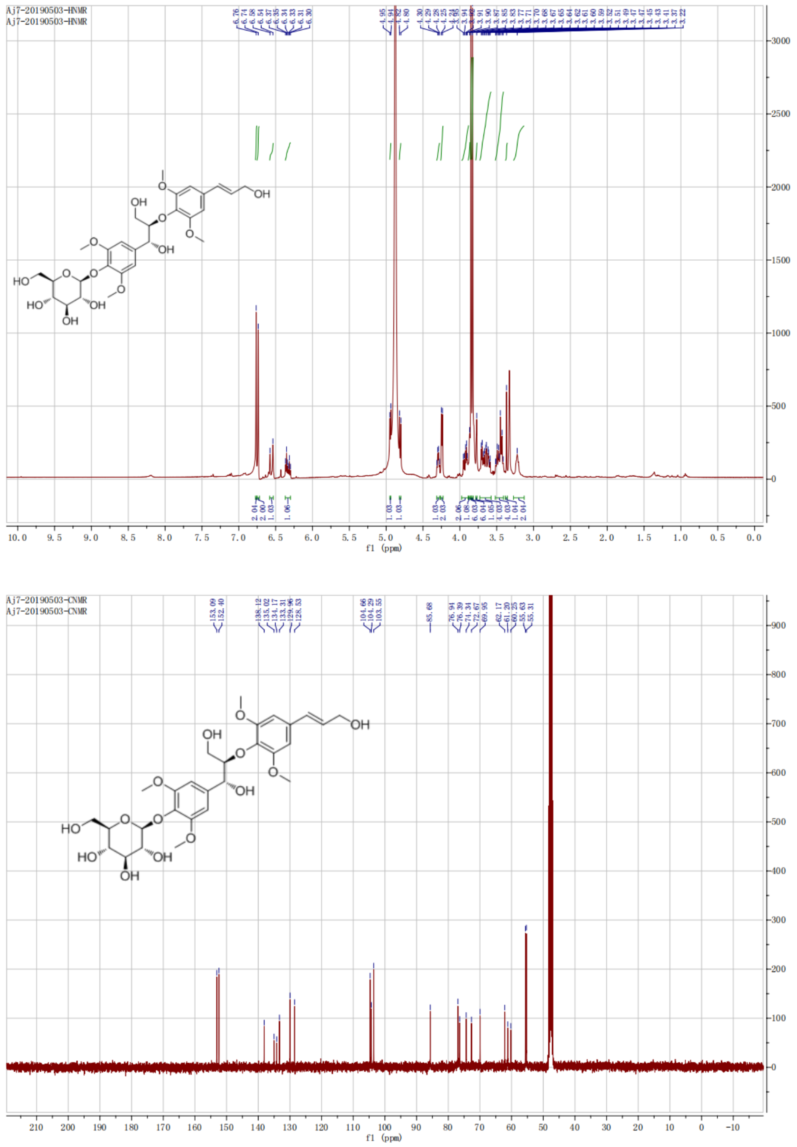

Fig. S8. ^{1}H-^{13}C NMR spectrum of compound **4**.

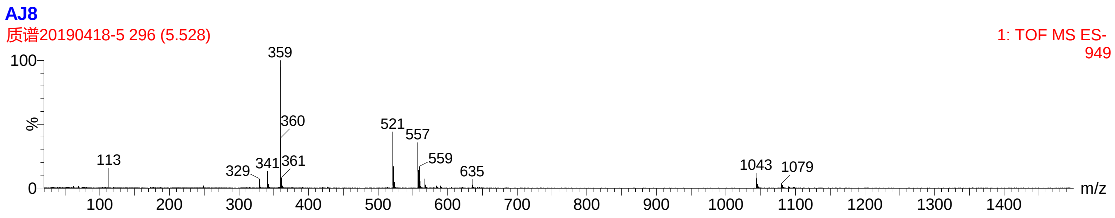

Fig. S9. ESI-MS spectrum of compound **5**.

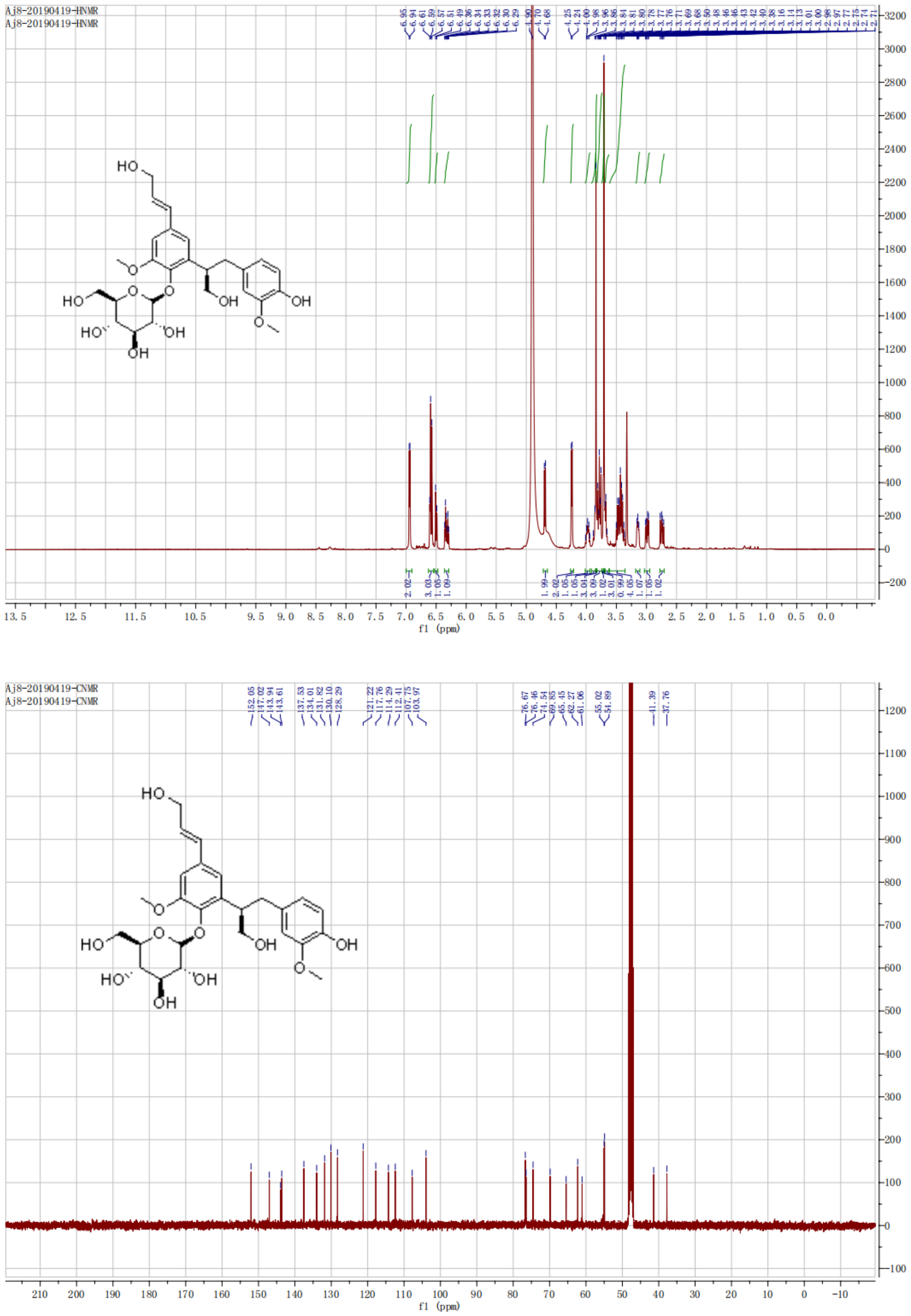

Fig. S10. ^{1}H-^{13}C NMR spectrum of compound **5**.
